# Comparison between acupuncture and cognitive behavioral therapy for primary insomnia: A protocol for systematic review and network meta-analysis

**DOI:** 10.1097/MD.0000000000020453

**Published:** 2020-05-22

**Authors:** Wei Peng, Ying Zhao, Yang Wang, Jun Wang, Qinghong Hao, Yang Tu, Tianmin Zhu

**Affiliations:** a Acupuncture & Tuina College; b Rehabilitation and Health Preservation College, Chengdu University of Traditional Chinese Medicine, Chengdu, Sichuan, China.

**Keywords:** acupuncture, cognitive behavioral therapy, network meta-analysis, primary insomnia, study protocol

## Abstract

**Background:**

Primary insomnia (PI) is a common disease affecting human health. As the side effects of drug therapy were revealed, people began to seek more safe and effective non-drug therapies. Cognitive behavioral therapy for insomnia (CBT-I) and acupuncture are 2 commonly used non-drug therapies. However, there are few comparative studies on the efficacy of these 2 therapies. Therefore, this study aims to compare the efficacy and safety of the 2 therapies through network meta-analysis.

**Methods:**

We will search the following electronic bibliographic databases: PubMed, EMBASE, The Cochrane Library, Web of Science, China National Knowledge Infrastructure, Chinese Biomedical Literature Database, Chongqing VIP database, and Wanfang database. Randomized controlled trials in which the intervention was acupuncture or CBT, and in which the control group was any of the above, western medicine or blank control, would be included. The primary outcome will be the changes of the Pittsburgh Sleep Quality Index, and the additional outcomes will include the changes in Insomnia Severity Index, quality of life, clinical effective rate and adverse events. Two independent authors will screen the literature in the above database, extract data and cross-check. Heterogeneity and inconsistencies are detected before using a network meta-analysis method based on frequency analysis. The risk of bias will be assessed in accordance with the Cochrane risk of bias tool, and the strength of the recommendations will be assessed by the Grading of Recommendations Assessment, Development and Evaluation.

**Ethics and dissemination:**

This network meta-analysis will provide a reference for clinicians and PI patients to choose a more appropriate non-drug regimen among multiple kinds of acupuncture or CBT-I therapies. This review does not require ethical approval and will be reported in a peer-reviewed journal.

**Trial registration number:**

PROSPERO CRD42020155327

## 1 Introduction

Primary insomnia (PI) is a common health disease characterized by difficulty in initiating or maintaining sleep, which is associated with daytime consequences, and lack of clear cause.^[1]^ When these insomnia symptoms persisted for at least 3 months, it could be defined as chronic.^[2]^ It is reported that there are about a fifth of the world's population has suffered from insomnia,^[3–5]^ and the incidence rate is increasing year by year.^[6–7]^ Recent research has found that insomnia could increase the risk of cardiovascular and cerebrovascular diseases, psychiatric diseases, and diabetes.^[8–11]^ Insomnia, therefore, has become a worldwide health problem that cannot be ignored.

Pharmacotherapy is the most commonly used treatment for PI because of convenience and effectiveness.^[12]^ However, these drugs are not perfect and still have some side effects. For example, a study from the United States found that frequent adverse events associated with drug therapy included inefficacy, drug resistance, amnesia, and nightmares, with inefficacy being the most common complaint.^[13]^ Therefore, non-drug therapy has been paid more and more attention in clinical practice. Cognitive behavioral therapy for insomnia (CBT-I) is currently preferred non-drug therapy without side effects.^[1]^ A systematic review found that CBT-I was effective in treating PI and preventing its recurrence.^[14]^

In recent years, growing evidence demonstrated that acupuncture was also a safe and effective nonpharmacological treatment in improving sleep quality and cognitive function.^[15–17]^ Acupuncture is a physical stimulation therapy that originated in China and widely used in the Asian.^[18]^ Acupuncture has been added to the guidelines for the diagnosis and treatment of insomnia in China.^[19]^ As far as we know, acupuncture is also a popular alternative medicine in the United States. Insomnia is among the top 10 acupuncture indications in the United States, according to an investigation of private clinics in 2018.^[20]^ Recent network systematic reviews have shown that several kinds of acupuncture methods were all effective and safe in improving the condition of patients with PI compared with drugs.^[21]^Although Many guidelines have recommended CBT-I as a first-line treatment for PI, most acupuncture studies rarely use it as a control group, often choosing either a positive drug or a placebo as a control group. There are only a handful of randomized controlled trials (RCTs) that directly compare acupuncture with CBT-I in the treatment of PI. For these reasons, there is no systematic review of direct comparisons between acupuncture and CBT-I.

Which non-drug treatment is the best choice for patients with PI between CBT-I and acupuncture? The answer is unclear, for lack of sufficient evidence. To solve the question, we will use a network meta-analysis (NMA) method that provides the possibility of comparing the indirect evidence. NMA can compare multiple interventions simultaneously and combine direct and indirect evidence to select the best treatment.^[22]^ In recent years, NMA has already been used successfully to compare different acupuncture methods for treating insomnia disorder,^[21]^ but few have focused on comparing it to CBT-I. Therefore, this systematic review and NMA based on high-quality RCTs data aims to analyze the comparison between different acupuncture therapies and CBT therapies and their safety and to calculate the effect size. Secondly, by ranking these non-drug regimens, we hope that the results of this study will provide a reference for clinicians and patients to choose the best non-drug treatment.

## 2 Methods

### 2.1 Design and registration

This systematic review and NMA will assess the comparative effectiveness and safety of acupuncture treatments and CBT-I treatments for PI. This protocol is drafted according to the Preferred Reporting Items for Systematic Review and Meta-Analysis Protocols^[23]^ and registered at PROSPERO (CRD42020155327). The final report will under the guidance of the PRISMA Extension Statement for Reporting of Systematic Reviews Incorporating Network Meta-Analyses of health care interventions^[24]^ and the Cochrane Collaboration Handbook for systematic reviews of interventions (V.5.1.0).

### 2.2 Ethics

Since our research is based on published research findings that have been reviewed by the Ethics Committee, this study does not require ethical approval.

### 2.3 Eligibility criteria

#### 2.3.1 Type of studies

All the RCTs of acupuncture and CBT-I in treating PI regardless of blinding and concealment will be included. However, only literatures written in English or Chinese will be included.

#### 2.3.2 Type of participants

Participants diagnosed with PI by either of the following diagnostic criteria will be included: diagnostic and statistical manual of mental disorders, international classification of sleep disorders, Chinese classification and diagnosis of mental diseases, as well as other accepted diagnostic criteria without gender, age, and ethnic origin restrictions.

#### 2.3.3 Type of interventions and comparators

Intervention: The experimental groups will be treated with acupuncture (include traditional acupuncture, or electroacupuncture, or auricular acupuncture, or scalp acupuncture, or warm acupuncture, etc), and CBT-I (include CT, BT, CBT).

Comparator: The control groups include drug treatment group and blank group. Or either of the described intervention compared with each other is also eligible.

#### 2.3.4 Outcome measures

The primary outcome will be the changes of the Pittsburgh Sleep Quality Index.^[25]^

The additional outcomes will include the following items: sleep score measured by Insomnia Severity Index^[26]^; quality of life obtained from the corresponding scale; clinical effective rate and adverse events,

#### 2.3.5 Exclusion criteria

Studies that do not have data available or cannot be extracted will be excluded.

### 2.4 Search strategy

We will search the following electronic bibliographic databases: English databases (PubMed, EMBASE, the Cochrane Library, Web of Science) and Chinese databases (China National Knowledge Infrastructure, Chinese Biomedical Literature Database, Chongqing VIP database, and Wanfang database) from their inception to October 1, 2019. We will also search for ongoing trial registers in the trial registry websites. At the same time, we will retrieve other potential articles manually in the reference list of retrieved studies to ensure the comprehensiveness of this research. There will be no restrictions on date limit, country, publication status, or year of publication.

We will use a combination of medical subject heading or keywords, and free-text terms with various synonyms. The medical subject heading or keywords used for the search will contain “acupuncture”, “insomnia”, “CBT-I”, “RCT” and synonymous words. The preliminary retrieval strategy for PubMed is presented in Table 1, which will be adjusted in accordance with specific databases.

**Table 1:**
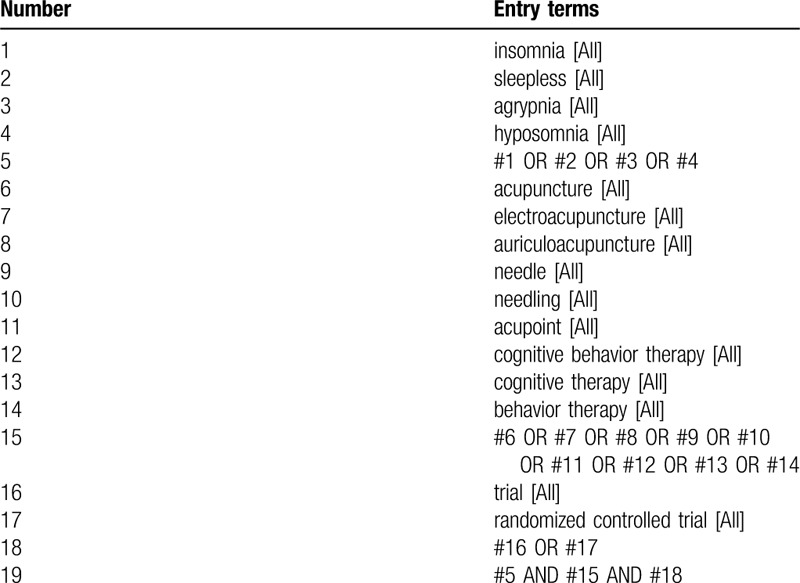
PubMed search strategy draft.

### 2.5 Selection of studies and data extraction

**Figure 1:**
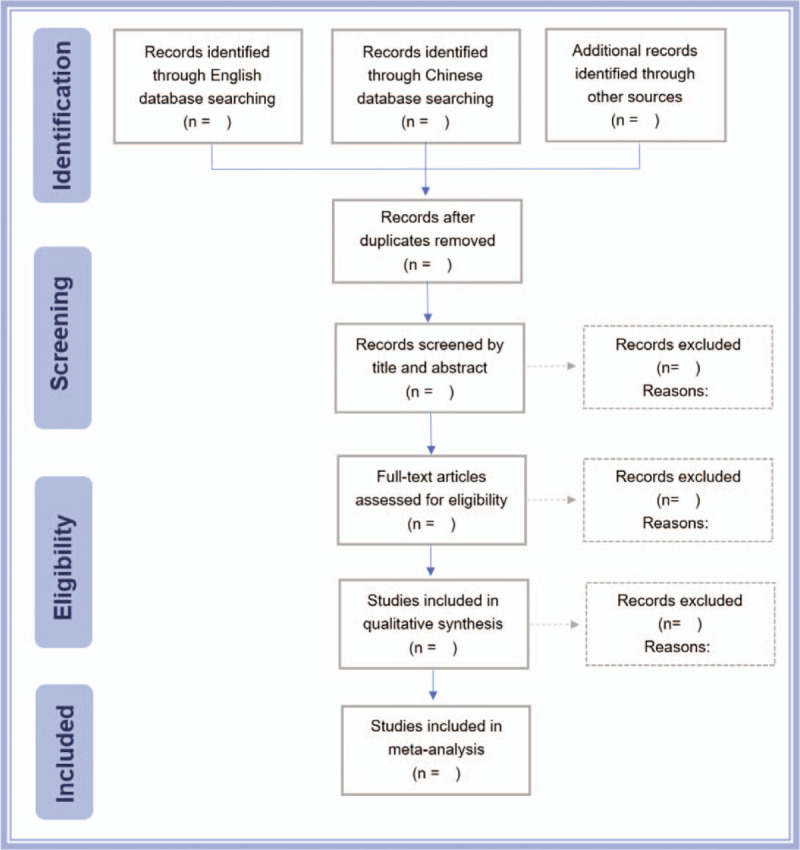
Flow chart of study selection.

Two reviewers (JW and QHH) will independently screen the literature in the above database, extract data, and cross-check. The retrieved articles will be imported into the Endnote software (version X9.2). They will screen these articles based on inclusion and exclusion criteria. The flow chart is displayed in Figure 1. If there were differences, they would be resolved through discussion or consultation with the third reviewer. After the screening, a unified form will present the contents of data extraction in Microsoft Excel (version 2016). If there is any missing data in the included literature, we will try to contact the original author by email or telephone to obtain the complete data.

The contents we want to extract are as follows:

(1) general information: include authors, journal, publication year, country, methods of randomization, and blinding;
(2) participants: include age, sex, duration of insomnia symptom;
(3) interventions and controls: include the type of therapy, the course of treatment, dosage forms, and clinical doses;
(4) outcomes: include the primary and secondary outcomes, measurement time points, and the proportion of adverse events.

### 2.6 Assessment of risk of bias

Two researchers (YZ and WP) will evaluate the risk of bias independently according to the Cochrane Collaboration's risk of bias tool.^[27]^ The following characteristics will be assessed: random sequence generation, allocation concealment, blinding of participants, and personnel, blinding of outcome assessment, incomplete outcome data, selective reporting, and other bias. Any disagreements will be resolved by discussion or discussed with another researcher (YT or QHH) if necessary.

### 2.7 Data synthesis and statistical methods

#### 2.7.1 Pairwise and network meta-analysis

Data analysis will be performed with the R software 3.6.1 (http://www.r-project.org/). We will use the “netmeta” package in R to complete the network meta-analysis. This package overcomes the difficulties of the software and package developed based on Bayesian statistical school in realizing the network meta-analysis^[28]^. We will perform network meta-analysis combining direct and indirect comparisons from the included RCTs.

For the direct comparisons, if there are more 2 RCTs, we will conduct a pairwise meta-analysis. For the results of indirect comparison, a network meta-analysis will be needed to adjust and mix results to improve accuracy and statistical efficacy. Continuous variable data will be presented as the standard mean difference with 95% confidence interval, whereas dichotomous data will be presented as odds ratio with 95% confidence interval. For direct and indirect comparisons, we will present the results through a network diagram.

#### 2.7.2 Assessment of heterogeneity

Before choosing fixed or random effect model to combine the effect size, we will run a heterogeneity test. The purpose of heterogeneity test is to check whether the results of individual studies are mergeable, and the test will be performed by calculating I^2^ statistic. If the value of I^2^ is less than or equal to 50%, the heterogeneity is small, and the fixed effect model will be used to combine the effect size. Conversely, if I^2^ > 50%, the source of heterogeneity was further analyzed. After excluding the influence of apparent clinical heterogeneity, the random effect model was used for meta-analysis.

#### 2.7.3 Subgroup and sensitivity analysis

If the heterogeneity is higher than 50%, we will conduct subgroup analysis and sensitivity analysis to explain the sources of heterogeneity. The subgroup analyses will be based on age, interventions, controls, and population area, while the sensitivity analyses based on research features or types.

#### 2.7.4 Assessment of inconsistency

Consistency hypothesis is an important hypothesis in network meta-analysis. It can assess the difference between direct comparative evidence and indirect comparative evidence. Inconsistency will be assessed by *Z* test when a loop is established among interventions.^[29]^

#### 2.7.5 Publication bias

We will also use funnel plots and Egger test to identify whether there is a small sample effect evaluation or potential publication bias.^[30]^ If the distribution of the points on both sides of the vertical line in the funnel plot is uniform, it means that there is no publication bias, and on the contrary, there is publication bias.

### 2.8 Grading the quality of evidence

The evidence quality will be evaluated by the Grading of Recommendations Assessment, Development and Evaluation.^[31]^ The quality of evidence in Grading of Recommendations Assessment, Development and Evaluation is classified as high, medium, low, and very low. Factors that reduce the quality of evidence include risk of bias, indirectness, inconsistency, imprecision, and publication bias. In addition, there are 3 factors that promote the level of evidence: large effect size, dose-effect relationship, and related confounding.

## 3 Discussion

We designed this network meta-analysis protocol on the basis of previous studies.^[22,32–33]^ Previous studies have found that auricular acupuncture had a greater effect on sleep duration in insomniacs than CBT.^[34–35]^ However, due to the small number of studies, the traditional systematic evaluation cannot reach a reliable conclusion. Whether acupuncture is better than CBT-I remains to be further studied. Treatments that are safe, effective, and have fewer side effects are more acceptable to patients. CBT-I and acupuncture have both been shown to be safe and effective non-drug therapies.^[32,36]^ Which is the best choice for patients? The network meta-analysis is beneficial in the absence of direct evidence. It provides an indirect comparison between the 2 treatments using a common control group. It can also rank a variety of acupuncture therapies with CBT, and help clinicians to choose a suitable non-drug therapy for insomnia patients.

## Author contributions

**Conceptualization:** Wei Peng, Ying Zhao, Tianmin Zhu.

**Data curation:** Jun Wang, Qinghong Hao.

**Formal analysis:** Wei Peng, Ying Zhao.

**Funding acquisition**: Tianmin Zhu.

**Investigation:** Wei Peng, Ying Zhao, Jun Wang, Qinghong Hao, Yang Tu.

**Methodology:** Wei Peng, Yang Wang.

**Supervision**: Tianmin Zhu.

**Writing – original draft:** Wei Peng.

**Writing – review & editing:** Ying Zhao, Yang Wang, Tianmin Zhu.
